# The Role of *Vaccinium Myrtillus* in the Prevention of Renal Injury in an Experimental Model of Ruptured Abdominal Aortic Aneurysm

**DOI:** 10.21470/1678-9741-2019-0121

**Published:** 2020

**Authors:** Şaban Ergene, Doğuş Hemşinli, Sedat Ozan Karakişi, Tolga Mercantepe, Levent Tumkaya, Adnan Yilmaz

**Affiliations:** 1 Department of Cardiovascular Surgery, Recep Tayyip Erdogan University, Faculty of Medicine, Rize, Turkey.; 2 Department of Histology and Embryology, Recep Tayyip Erdogan University, Faculty of Medicine, Rize, Turkey.; 3 Department of Medical Biochemistry, Recep Tayyip Erdogan University, Faculty of Medicine, Rize, Turkey.

**Keywords:** Aorta, Abdominal, Renal Artery, Laparotomy, Malondialdehyde, Sodium Chloride, Oxidative Stress, Apoptosis

## Abstract

**Objective:**

To examine the biochemical and histopathological renal effects of ischemia/reperfusion (I/R) injury using a ruptured abdominal aortic aneurysm (RAAA) model in rats and to investigate the potential protective effects of whortleberry (*Vaccinium myrtillus*).

**Methods:**

Thirty-two male Sprague-Dawley rats were randomly assigned into four groups - control, sham (I/R+glycerol), I/R, and I/R+whortleberry. Midline laparotomy alone was performed in the control group. Atraumatic abdominal clamps were attached under anesthesia to the abdominal aorta beneath the level of the renal artery in the groups subjected to I/R. Sixty-minute reperfusion was established one hour after ischemia. The sham group received five intraperitoneal doses of glycerol five days before I/R. The I/R+whortleberry group received a single intraperitoneal 50 mg/kg dose diluted with saline solution five days before I/R. All animals were finally euthanized by cervical dislocation following 60-min reperfusion.

**Results:**

Increases were observed in malondialdehyde (MDA) levels and tubular necrosis scores (TNS) in thin kidney tissues and in numbers of apoptotic renal tubule cells, together with a decrease in glutathione (GSH) levels, in sham and I/R groups. In contrast, we observed a decrease in MDA levels, TNS, and numbers of apoptotic renal tubule cells, and an increase in GSH levels with whortleberry treatment compared to the I/R group.

**Conclusion:**

Our findings suggest that whortleberry may be effective against acute kidney injury by reducing oxidative stress and apoptosis.

**Table:** 

Abbreviations, acronyms & symbols	
ANOVA	= Analysis of variance
GSH	= Glutathione
HPS	= Histopathological score
HSD	= Honestly significant difference
IP	= Intraperitoneal
I/R	= Ischemia/reperfusion
MAP	= Mean arterial pressure
MDA	= Malondialdehyde
RAAA	= Ruptured abdominal aortic aneurysm
ROS	= Reactive oxygen radicals
SOD	= Superoxide dismutase
SPSS	= Statistical Package for the Social Sciences
TNS	= Tubular necrosis scores
Wb	= Whortleberry

## INTRODUCTION

Ruptured abdominal aortic aneurysm (RAAA) is one of the most important emergencies in cardiovascular surgery, with mortality rates of 40-75%^[1]^. Hemorrhagic shock arises from lower body ischemia and reperfusion develops in association with aortic clamping during surgical treatment of RAAA^[2]^. Bleeding during surgical treatment must be stopped immediately and hemodynamics rapidly restored by applying cross-clamps to the aorta^[3]^. Declamping shock may occur in association with reperfusion of the lower body when the repair is completed and the aortic clamps are removed. In addition, reperfusion injury then develops, and the resulting reactive oxygen radicals (ROS) further exacerbate systemic inflammatory response syndrome. Distant organ damage occurring when hemodynamics cannot be rapidly adjusted and in the presence of severe reperfusion injury increases perioperative mortality and morbidity^[1-3]^.

Acute kidney injury associated with ischemia/reperfusion (I/R) injury during RAAA surgery is an important clinical problem with a mortality rate exceeding 50%^[4,5]^. Although the damage mechanism is unclear, in addition to occlusion of the aorta, reperfusion has also been reported to be capable of causing acute kidney injury through several complex mechanisms, including ROS, neutrophil infiltration, and the release of inflammatory mediators^[6-8]^. ROS cause cellular damage through their deleterious effects on antioxidant defense mechanisms by reducing levels of glutathione (GSH) peroxidase and increasing those of malondialdehyde (MDA)^[9]^. In addition, recent studies have reported that I/R injury leads to caspase-dependent apoptosis associated with increased ROS^[10]^. Although several caspase cascades are involved in apoptosis, Caspase-3 is regarded as an irreversible terminal event in the activation of the apoptotic mechanism and plays a key role in ROS-related apoptosis. On the other hand, ROS cause cellular damage by resulting in adverse effects on antioxidant defense mechanisms^[11,12]^. Antioxidants, such as catalase, glutathione peroxidase, ceruloplasmin, and superoxide dismutase (SOD), protect cells against lipid peroxidation by reducing GSH levels^[6,7,13]^. MDA is a product of unsaturated fatty acid breakdown and a marker of lipid peroxidation in tissue^[14]^.

Whortleberry (*Vaccinium myrtillus* L.) exhibits antioxidant and anti-inflammatory capacities due to its high anthocyanin, flavonoid, and phenolic acid content. It has also been reported to suppress enzymatic processes that cause oxidative damage by raising MDA and GSH levels and to effectively eliminate ROS^[15-17]^.

The purpose of our study, conducted using an RAAA model in rats, was to examine the biochemical and histopathological effects of I/R injury on the kidney and to investigate the potential protective effect of whortleberry.

## METHODS

### Animals

Thirty-two male Sprague-Dawley rats (250±50 g, 3-5 months old) were used in the study. All animals were fed and housed at 22±2°C room temperature and 55-60% humidity in a 12-h light:12-h dark cycle in the experimental animals’ unit of the Recep Tayyip Erdoğan University, Medical Faculty, Experimental Animals Application Center (Rize, Turkey). All animals received humane care according to the criteria outlined in the ‘Guide for the Care and Use of Laboratory Animals’ prepared by the National Academy of Sciences and published by the National Institutes of Health. The study was approved by the Recep Tayyip Erdoğan University Animal Ethical Committee (Rize, Turkey).

### Experimental Groups

The animals used in this study were randomly divided into four groups containing eight members each - control, sham (solvent, glycerol+I/R), I/R, and I/R+whortleberry. No surgical procedure was applied to the control or sham groups other than aortic exploration. Sham, I/R, and whortleberry groups were exposed to shock for 60 min, ischemia for 60 min, and reperfusion for 120 min.

### Aortic Clamping Technique

The RAAA model applied to the rats in the sham (glycerol+I/R) and whortleberry groups in our study was designed on the basis of previous studies^[3,18]^. All groups fasted for 12 h before the surgical procedures. They were then placed on the operating table and immobilized with intraperitoneal (IP) xylazine (5 ml/kg) and ketamine (40 mg/kg) anesthesia. The abdominal area on which the surgical procedure was to be performed was shaved under sterile conditions and washed with povidone-iodine solution. The area was then opened with median laparotomy. The right carotid artery was cannulated with a 22G Intracath for arterial pressure monitoring, and the left internal jugular vein was similarly cannulated for blood and fluid replacement via surgical exploration. The arterial line was attached to a monitor with a three-way faucet and a transducer set, and invasive pressure monitoring was maintained throughout the experiment. At the end of the catheterization procedure, shock was induced through controlled blood collection from the carotid artery into an injector containing 500 units (U) of heparin until a value of mean arterial pressure (MAP) ≤ 50 mmHg was achieved in such a way as to simulate aneurysm rupture and hemorrhagic shock in subjects from all groups, except for the control group. Blood was stored at room temperature for use in blood resuscitation. The shock was maintained for 60 min once a value of MAP ≤ 50 mmHg had been attained. During this procedure, blood was again collected as necessary to ensure the maintenance of MAP ≤ 50 mmHg, and the amounts and timings involved were recorded. The abdominal aorta was explored with median laparotomy toward the end of the shock stage. Anticoagulation was established (laparotomy + ischemia + resuscitation stage) with intravenous administration of 100 IU of heparin to all subjects at the end of exploration. When the 60-min shock stage was completed, ischemia was induced in all subjects with the attachment of bulldog clamps to the infrarenal abdominal aorta artery and iliac bifurcation. Resuscitation commenced at the moment the clamps were attached by returning half the blood previously collected and stored at room temperature via the internal jugular vein (the ischemic process in the model simulates surgical treatment, in other words, stopping the bleeding with an aortic clamp, and surgical reconstruction). The rats in the sham group received 1 ml of glycerol, one dose per day, for five days before ischemia. Rats in the whortleberry treatment group received 100 ml/kg of IP *Vaccinium myrtillus*, one dose per day, for five days before ischemia^[16,19]^. At the end of the experimental period, all rats were sacrificed by exsanguination from the carotid artery. One part of the extracted kidney tissues was frozen and stored at -80°C for biochemical analyses, while the other part was fixed in 10% neutral formalin for histopathological investigation.

### Biochemical Investigations

#### Homogenate Preparation

Renal tissue specimens were washed in cold phosphate buffer, after which cold phosphate buffer was added to the kidney tissue specimens to a volume twice that of the renal tissue. All specimens were homogenized for one minute at 30 Hertz. The homogenized tissues were then centrifuged at +4°C and at 3000 g for 15 min, after which the supernatant part was removed for biochemical analysis.

#### Renal MDA and GSH Levels Measurement

The Ellman method was used to measure renal GSH levels. This method is based on the principle of spectrophotometric measurement of the color formed by free sulfhydryl groups with Ellman’s reagent in liver homogenate^[20]^.

MDA measurement was performed using the method described by Draper and Hadley. MDA, the final product of lipid peroxidation, yields a pink complex giving maximum absorbance at 532 nm by reacting with thiobarbituric acid^[14]^.

### Histopathological Analysis Procedure

Kidney tissue specimens were washed in phosphate buffer (pH 7.4, Sigma-Aldrich, Germany) and then fixed in 10% phosphate-buffered formalin (Sigma-Aldrich, Germany) for 36 h. Following fixation, specimens were subjected to routine histological procedures, embedded in paraffin blocks (Merck Darmstadt, Germany), and sliced into 4-5 µm thick sections with a microtome (Leica RM2525, Lecia, Germany). Finally, the sections were stained with Harris hematoxylin and eosin G (H&E; Merck, Darmstadt, Germany).

### Immunohistochemistry Analysis Procedure

Caspase-3 (1:200, rabbit polyclonal Caspase-3, Abcam, UK) placed onto positively charged slides was used as the primary antibody for the determination of apoptotic renal tubule cells, together with kits containing an appropriate secondary antibody (goat anti-rabbit IgG H&L [HRP], ab205718, Abcam, UK).

Kidney tissue sections, 1-3 µm in thickness and cut using a microtome, were placed onto positively charged slides and subjected to deparaffinization and antigen retrieval procedures. Next, following incubation with primary and secondary antibodies, in line with the manufacturer’s instructions, 3,3-diaminobenzidine tetrahydrochloride (Sigma Chemical, St. Louis, MO, USA) was applied. Finally, sections were counterstained with Harris hematoxylin (Merck, Darmstadt, Germany).

### Semiquantitative Analysis

Renal damage grade scoring was performed using the methods described by Jeong Sung et al.^[21]^ (Table 1). Thirty randomly selected different areas on each prepared were evaluated by two histopathologists blinded to the experimental groups and macroscopic description (TM and LT).

**Table 1:** Tubular necrosis score classification by Sung MJ et al.^[21]^

Score	Percentage (%)
Impairment of brush margin structure in proximal tubules	
0	No damage
1	≤ 10%
2	10-25%
3	26-75%
4	≥ 75%
Atypical apical membranes (blebbing)	
0	No damage
1	≤ 10%
2	10-25%
3	26-75%
4	≥ 75%
Loss of connections between tubular epithelial cells or through the basal membrane (debris accumulation within the lumen)	
0	No damage
1	≤ 10%
2	10-25%
3	26-75%
4	≥ 75%

### Statistical Analysis

Data obtained from semiquantitative and biochemical analyses were analyzed using the Statistical Package for the Social Sciences (SPSS) software (IBM, NY, USA), version 18.0. Non-parametric data obtained from semiquantitative analysis were calculated as median±standard deviation, and differences between groups were analyzed using the Kruskal-Wallis test, followed by the Tamhane’s T2 test. Parametric data obtained from biochemical analysis were calculated as mean±standard deviation, and differences between groups were subjected to one-way analysis of variance (ANOVA) followed by the Tukey’s honestly significant difference test (or Tukey’s HSD test). *P* values < 0.05 were regarded as significant.

## RESULTS

### Biochemical Results

MDA levels in kidney tissues from the sham and I/R groups were significantly higher than those in the control group (*P*=0.01 and *P*=0.00, respectively) (Table 2). In contrast, we observed a significant decrease in MDA levels in the whortleberry group compared to the control group (*P=*0.015) (Table 2).

**Table 2:** Biochemical analysis results (mean±standard deviation).

Groups	MDA (µmol/g) in tissue	GSH (µmol/g) in tissue
Control	0.32±0.13	34.29±1.34
Sham	0.35±0.14^a^	29.51±1.55^d^
I/R	0.36±0.17^b^	30.31±1.43^e^
I/R+whortleberry (Wb)	0.33±0.19^c^	33.59±2.66^f^

Tukey's honestly significant difference test (or Tukey's HSD test):

a P=0.01 control group compared to the glycerol group,

b P=0.00 control group compared to the I/R group,

c *P*=0.015 I/R group compared to the I/R+Wb group,

d v=0.00 control group compared to the glycerol group,

e *P*=0.02 control group compared to the I/R group, and

f *P*=0.13 I/R group compared to the I/R+WB group.

I/R group’s kidney GSH levels decreased compared to the control group (*P*=0.00) (Table 2). Similarly, I/R+glycerol group’s kidney tissue GSH levels were lower than those of the control group (*P*=0.02) (Table 2). In contrast, GSH levels increased significantly in the whortleberry group compared to the control group (*P*=0.013) (Table 2).

### Light Microscopy Results

Renal corpuscles and proximal and distal tubules in kidney sections from the control group exhibited a normal architecture, and brush borders in proximal tubules were also normal in structure (median tubular necrosis scores [TNS]: 0.00±0.52). Sections from the sham group exhibited atypical glomerules with numerous necrotic tubules and loss of brush margins. We also determined vascular congestion in interstitial areas and areas of inflammation (median histopathological score [HPS]: 13.50). In sections from the I/R group, we observed an atypical glomerular structure and numerous necrotic tubules with loss of brush margins. In addition, we determined debris depositions in the lumens of necrotic tubules. We also observed vascular congestion and inflammation in interstitial spaces (median HPS: 15.50). We observed a decrease in necrotic tubules in the whortleberry group compared to the I/R group, while the intertubular spaces were typical in appearance (median HPS: 1.50).

### Immunohistochemical Results

**Fig. 1:**
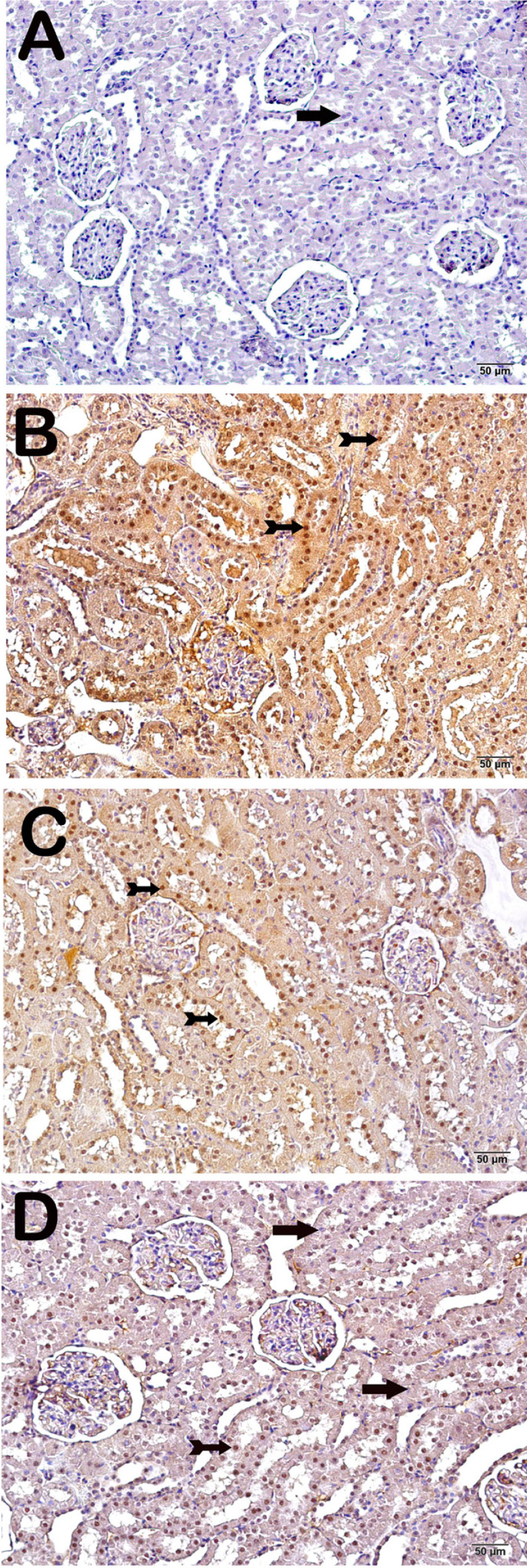
Light microscopic image of immunohistochemical staining. Caspase-3. A (´20): sections from the control group show a normal structure in proximal and distal tubule epithelial cells (arrow) (Caspase-3 positivity score=0.00±0.46). B (´20): sham group sections showing intense Caspase-3 positivity in proximal and distal tubule epithelial cells (arrow) (Caspase-3 positivity score=3.00±0.53). C (´20): sections from the I/R group showing intense Caspase-3 positivity in proximal and distal tubule epithelial cells (Caspase-3 positivity score=3.50±0.35). D (´20): sections from the whortleberry treatment group showing a typical structure in proximal and distal tubule epithelial cells, with slight Caspase-3 positivity in epithelial cells (tailed arrow) (Caspase-3 positivity score=1.00±0.52). I/R=ischemia/reperfusion

Table 3 shows the grading of Caspase-3 staining positivity scores. Examination of kidney tissue sections from the sham and I/R groups revealed significantly higher Caspase-3 positivity in proximal and distal tubule epithelial cells compared to the control group (*P*=0.00 and *P*=0.00, respectively) (Table 4). In contrast, Caspase-3 scores in proximal and distal tubule epithelial cells decreased significantly in the whortleberry group compared to the I/R group (*P*=0.03 and *P*=0.00, respectively) (Figure 1 and Table 4).

**Table 3:** Grading of Caspase-3 staining positivity scores.

Grade	
1	None (< 25%)
2	Mild (25-50%)
3	Moderate (50-75%)
4	Severe (> 75%)

**Table 4:** Caspase-3 positive grade scores data (median±standard deviation).

Group	Scores
Control	0.00±0.46
Sham	3.00±0.53^a^
I/R	3.50±0.35^b^,^c^
I/R+whortleberry	1.00±0.52

Kruskal-Wallis test/Tamhane's T2 test:

a *P*=0.00 compared to the control group,

b *P*=0.00 compared to the I/R group,

c *P*=0.03 compared to the control group.

### Semiquantitative Analysis

**Fig. 2:**
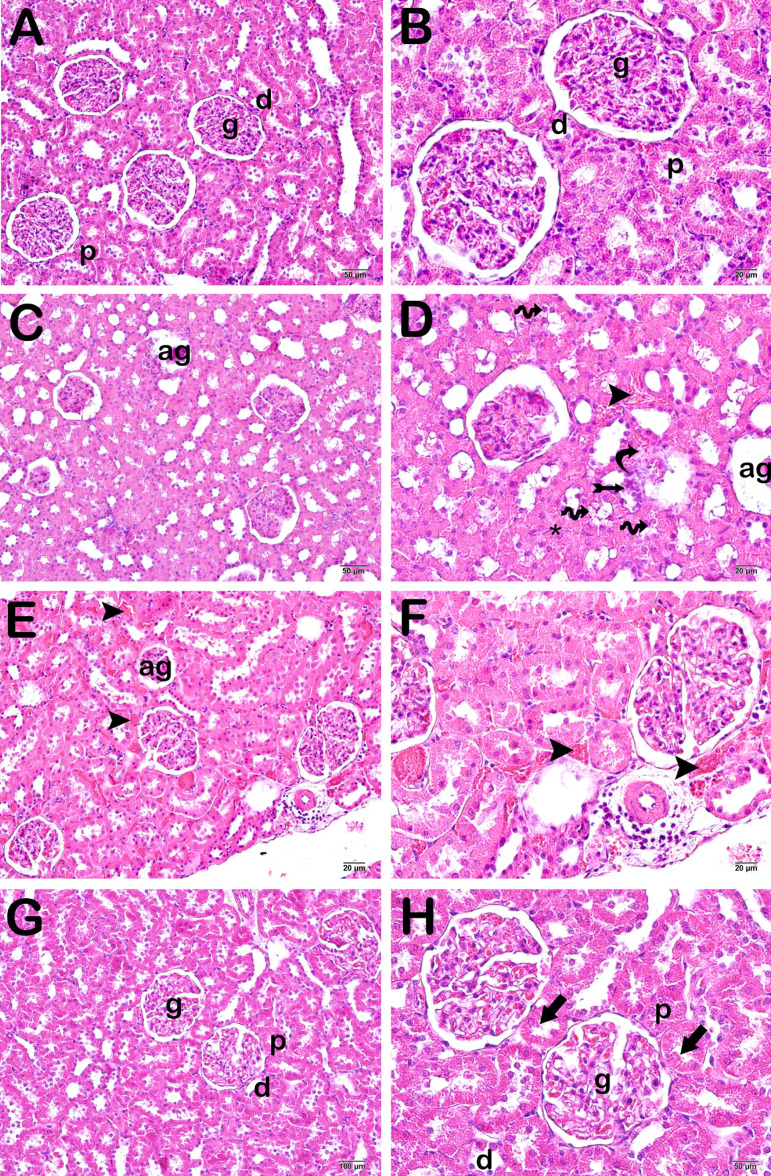
Microscopic image of kidney tissue. H&E staining of proximal tubule (p), distal tubule (d), glomerule (g), and brush margin (arrow). A (´20) and B (´40): normal glomerular (g), proximal tubule (p), and distal tubule (d) structures in control group sections. The brush margin structure (arrow) in the proximal tubules is clearly defined (TNS=1.00±076). C (´20) and D (´40): sections from the sham group show diffuse atypical glomerules (ag) and numerous necrotic tubule epithelial cells with loss of brush margins (spiral arrow). Vascular congestion (arrowhead) can be seen in interstitial spaces (curly arrow), together with inflammation (tailed arrow) (TNS=8.00±0.69). E (´10) and F (´20): sections from the I/R group show an atypical glomerulus (ag) structure and debris accumulation (star) in the tubular lumen. Numerous atypical proximal tubules with loss of brush margins (arrow) can also be seen. Leukocyte infiltration can be seen in interstitial spaces (tailed arrow) (TNS=8.00±1.35). G (´20) and H (´40): sections from the whortleberry group show glomerules (g) and proximal tubules (p) with a normal brush margin structure (TNS=2.00±0.49). I/R=ischemia/reperfusion; TNS=tubular necrosis score

TNS scores increased significantly in the sham and I/R groups compared to the control group (*P*=0.00) (Table 5 and Figure 2). However, TNS scores decreased significantly in the whortleberry group compared to sham and I/R groups (*P*=0.00 for both) (Table 5 and Figure 2).

**Table 5:** Tubular necrosis scores (TNS) analysis result data (median±standard deviation).

Groups	Brush margin damage score	Atypical apical membrane score (blebbing)	Debris accumulation within the lumen score	TNS
Control	0.00±0.29	0.00±0.49	0.00±0.35	1.00±076
Sham	3.00±0.585^a^	3.00±0.54^a^	2.50±0.35^a^	8.00±0.69^a^
I/R	3.00±0.38^a^	2.00±0.54^a^	3.00±0.54^d^	8.00±1.35^a^
I/R+whortleberry	1.00±0.69^b^	1.00±0.76^c^	0.00±0.35	2.00±0.49^b^

Kruskal-Wallis test/Tamhane's T2 test:

a *P*=0.00 compared with the control group,

b *P*=0.00 compared with the I/R group,

c *P*=0.03 compared with the I/R group,

d *P*=0.012 compared with the control group.

## DISCUSSION

Acute kidney injury occurring in association with perfusion disorder deriving from hemorrhagic shock and I/R in the surgical treatment of RAAA and intensive care follow-up is a significant clinical problem that can result in high mortality rates^[6,10,22]^. Deriu et al.^[22]^ reported that short-term reperfusion can protect the right and left renal arteries against acute kidney injury developing in association with abdominal aortic aneurysm^[22]^. Studies have also reported that abdominal aortic aneurysm can result in acute kidney injury by causing the production of free oxygen radicals^[23]^. The purpose of this study was, therefore, to use whortleberry, with its known antioxidative effects, in the treatment of oxidative stress occurring in association with free oxygen radicals in acute kidney injury caused by hemorrhagic shock in the surgical treatment of RAAA.

In their study involving an infrarenal aortic clamping model, Ulus et al.^[7]^ reported vascular congestion in interstitial spaces, but no pathological changes in the kidney. In contrast, Cüre et al.^[6]^ reported an atypical Bowman’s capsule and edematous areas resulting from shedding in renal tubule epithelial cells, together with debris accumulation in the tubular lumen as a result of abdominal aorta cross-clamping. They also reported extensive edema in intertubular spaces and areas of infiltration^[6]^. Similarly, in our study, we observed atypical renal corpuscles, necrotic renal tubules, and inflammation and vascular congestion in intertubular spaces. Additionally, we also observed losses in the brush borders of proximal tubule epithelial cells. However, we observed no edema in intertubular spaces.

Abdominal aortic cross-clamping model studies have reported that although the damaged mechanism giving rise to acute kidney injury is not yet fully understood, it causes oxidative stress in association with free oxygen radicals (ROS) production^[5,24]^. Oyar et al.^[25]^ reported that abdominal aortic cross-clamping increased MDA levels in kidney tissue. To the best of our knowledge, no studies involving abdominal aorta cross-clamping models have investigated GSH levels in kidney tissue, although Singh et al.^[26]^ have reported a decrease in GSH levels in kidney tissue following the application of renal I/R. Similarly, we observed that aortic cross-clamping increased MDA levels while reducing GSH levels in kidney tissue.

Studies involving both abdominal and renal I/R models have reported that I/R causes apoptosis in renal tubule cells^[11,27]^. In their study using an aortic cross-clamping model in rats, Cüre et al.^[10]^ reported an increase in Caspase-3 positivity in apoptotic renal cells following I/R application. Similarly, in the present study, we observed intensive Caspase-3 positivity in proximal and distal tubule epithelial cells.

Previous studies have reported that abdominal I/R causes apoptosis in renal cells in addition to an increase in ROS^[12,28]^. In their study involving an aortic cross-clamping model in rats, Oyar et al.^[25]^ showed that in addition to increasing MDA levels in kidney tissue, I/R also led to apoptosis in renal tubule cells by increasing activity of Caspase-3, one of the caspases involved in the irreversible terminal stage of apoptosis. Similarly, we determined an increase in MDA levels in kidney tissue and an increase in Caspase-3 in renal tubule epithelial cells following I/R based on an aortic clamp model. We also observed a decrease in renal GSH levels following I/R application.

Whortleberry is a member of the bilberry (*V. myrtillus*) family, with a natural anthocyanin pigment in flavylium cation form^[15]^. In addition to its antioxidant properties, whortleberry has also been reported to possess anti-inflammatory and anticarcinogenic characteristics^[17,20,29]^. Various studies have investigated the antioxidant property of whortleberry on tissues such as the liver, kidneys, lungs, and testes, but we found no studies of its effects on I/R^[30]^. However, Ziberna et al.^[31]^ reported that while bilberry anthocyanins exhibited powerful cardioprotective activity at low concentrations, it exacerbated I/R injury at high concentrations^[31]^. Bao et al.^[29]^ reported that whortleberry reduced oxidative stress and the degree of kidney injury by lowering MDA levels in renal tissue. However, to the best of our knowledge, no previous studies have investigated the effects of whortleberry on GSH levels in kidney tissue. Additionally, Eren et al.^[19]^ reported that whortleberry reduced oxidative stress and kidney injury by increasing the total antioxidant status in renal tissue. Similarly, in the present study, we determined that it lowered MDA levels that had previously risen following I/R application, while raising previously lowered GSH levels.

In addition to these findings, there are several limitations to our study. In order to avoid misinterpretation of the effects on renal tissue of the glycerol used as a solvent during the whortleberry preparation stage, we endeavored to reduce such error to a minimum by applying glycerol to healthy rats. In addition, oxidative stress should also be evaluated using other oxidant and antioxidant enzymes and molecules. Moreover, intracellular calcium levels should be separately assessed for analysis of apoptosis and free oxygen radicals.

## CONCLUSION

This study may lead to a new perspective in terms of the oxidative stress developing in association with lipid peroxidation induced in kidney tissue by abdominal aortic I/R. We also observed that it caused apoptosis in renal tubular epithelial cells. On the other hand, whortleberry protected against the kidney injury resulting from I/R by reducing lipid peroxidation and apoptosis. For a better understanding of the injury developing in association with aortic clamping, our study now needs to be supported by further studies investigating proinflammatory cytokines in particular, as well as other ROS enzymes and molecules.

**Table:** 

Author's roles & responsibilities	
SE	Substantial contributions to the conception or design of the work; or the acquisition, analysis, or interpretation of data for the work; final approval of the version to be published
DH	Substantial contributions to the conception or design of the work; or the acquisition, analysis, or interpretation of data for the work; final approval of the version to be published
SOK	Substantial contributions to the conception or design of the work; or the acquisition, analysis, or interpretation of data for the work; final approval of the version to be published
SOK	Substantial contributions to the conception or design of the work; or the acquisition, analysis, or interpretation of data for the work; drafting the work or revising it critically for important intellectual content; final approval of the version to be published
LT	Substantial contributions to the conception or design of the work; or the acquisition, analysis, or interpretation of data for the work; drafting the work or revising it critically for important intellectual content; final approval of the version to be published
AY	Substantial contributions to the conception or design of the work; or the acquisition, analysis, or interpretation of data for the work; drafting the work or revising it critically for important intellectual content; final approval of the version to be published
